# FDG PET-CT Finding in Bilateral Renal and Bone Involvement of Diffuse Large B-Cell Lymphoma

**DOI:** 10.4274/mirt.98608

**Published:** 2014-10-05

**Authors:** Yusuf Ziya Tan, Sabire Yılmaz, Meftune Özhan, Metin Halaç

**Affiliations:** 1 Çanakkale Onsekiz Mart University, Department of Nuclear Medicine, Çanakkale, Turkey; 2 Istanbul University Cerrahpasa Faculty of Medicine, Department of Nuclear Medicine, İstanbul, Turkey

**Keywords:** non-Hodgkin lymphoma, diffuse, large B-cell, Lymphoma, 18F-FDG, positron-emission tomography / computed tomography

## Abstract

Thirty-six year old male patient with pathological fracture of the left tibia underwent intramedullary and soft tissue curettage. The histopathological examination revealed diffuse large B cell lymphoma. The patient underwent F18-FDG PET-CT scanning for initial staging. FDG PET-CT scan revealed hypermetabolic lesions at the left tibia and in bilateral kidneys. After the systemic chemotherapy and local radiotherapy to the tibia, repeated FDG PET/CT scan showed improvement of the previous hypermetabolic lesions, suggesting good response to therapy. Bone and renal involvement is an uncommon variant of diffuse large B-cell lymphoma and FDG PET-CT is an useful whole body imaging modality in these cases.

## Abstract (tr)

Otuz altı yaşındaki erkek hastaya sol tibiasında patolojik kırık nedeniyle yapılan intramedüller ve yumuşak doku küretaj biyopsisi sonucu diffüz büyük B-hücreli lenfoma gelmesi üzerine, başlangıç evrelemesi için F18-FDG PET-BT incelemesi uygulandı. FDG PET-BT incelenmesinde sol tibia distalinde ve bilateral böbreklerde hipermetabolik tutulum izlendi. Sistemik kemoterapi ve sol tibiaya lokal radyoterapi yapıldıktan sonra tekrar edilen FDG PET-BT incelemesinde önceki hipermetabolik tutulumların düzeldiğinin görülmesi iyi yanıt varlığını düşündürdü. Kemik ve böbreklerde nadir tutulum gösteren diffüz büyük B-hücreli lenfomalarda FDG PET-BT tüm vücut görüntüleme yararlı bir tekniktir.

## INTRODUCTION

Non-hodgkin lymphomas (NHL) are grouped as nodal and extranodal according to the localization. Extranodal involvement constitute 25%-40% of all NHL cases and commonly observed among diffuse large B cell lymphoma (DLBCL) and follicular lyphoma patients (1). The most common extranodal localizations are skin, head-neck, stomach, spleen, lungs and bones. CT imaging has been used for staging and follow-up of these patients. Recently, it has been reported that FDG PET/CT scan is superior to CT scan especially in staging, follow-up and evaluation of response to treatment in patients with extranodal involvement such as mucosa, cortical bones, bone marrow, lungs, pleura and gastrointestinal system (2). Here, we describe an unusual case of DLBCL with concurrent involvement of bone and kidneys which were revealed by FDG PET/CT.

## CASE REPORT

**Figure 1:**
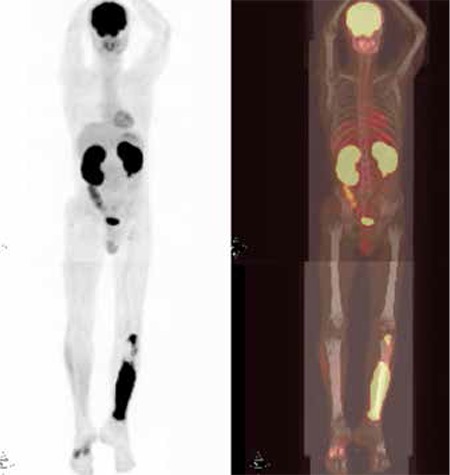
Thirty six-year-old male patient with pathologic fracture of the lefttibia underwent intramedullary curettage. Biopsy confirmed DLBCL. Patientunderwent FDG PET/CT scanning for staging. Maximum intensity projection(MIP) (left) and FDG PET/CT fusion (right) images demonstrated very intenseFGD accumulation (SUVmax:17.1) extending to soft tissue at the 2/3 distalpart of the left tibia and intense tracer uptake at the proximal metaphysisof the left tibia which were both compatible with disease involvement.Additionally, there was very intense diffuse FDG uptake in bilateral enlargedkidneys which was suspicious for primary disease involvement.

Thirty six-year-old male was presented with left tibial mass for the last 6 months. MRI showed contour irregularity and cortical thinning at the diaphysis of the left tibia and 1.5 cm severe periostal reaction at the same region. On follow-up, pathologic fracture of the left tibia occured. The intramedullary and soft tissue curettage of the left tibia, performed 3 months after the fracture, confirmed high grade diffuse large B-cell lymphoma. There is no past medical and genitourinary history. Laboratory analysis showed a normal complete blood count, normal urine analysis, and normal basic metabolic panel. Patient was reffered to our PET/CT unit for initial staging. FDG PET/CT scan showed a very intense FDG accumulation (SUVmax: 17.1) at the distal 2/3 of the left tibia extending to neighbouring soft tissue and proximal metaphysis of the left tibia which were compatible with primary disease involvement. Additionally, there was diffuse highly intense FDG uptake in the bilateral hyperplastic kidneys which was consistent with disease involvement (Figure 1). The possibility of nephrotoxicity was excluded because of normal laboratory examination and no drug history.

**Figure 2:**
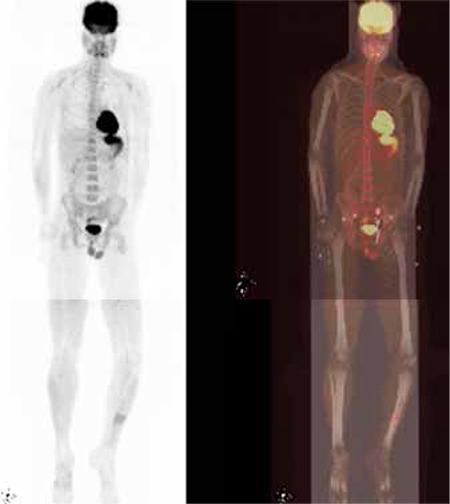
MIP (left) and PET/CT fusion (right) images of repeated FDG PET/CT scan after systemic chemotherapy and external local radiotherapy to theleft tibia. The uptake in bilateral enlarged kidneys, shown in previous PET/CTscan, was completely regressed whereas very intense accumulation at theleft tibia showed near complete regression. Additionally, there was slightlyincreased heterogenous FDG uptake in the fracture line at the middle partof left tibial diaphysis consistent with inflammatory process.

Patient underwent local external radiotherapy to the left tibia and systemic chemotherapy. Four months after the treatment, repeated FDG PET/CT scan revealed complete resolution of the renal lesions, indicative of complete remission. There was near-complete remission in the left tibia lesion. Middle part of left the tibial diaphysis which showed slightly increased FDG uptake in the fracture line was considered as an inflammatory process (Figure 2).

**Literature Review and Discussion**

Non-hodgkin lymphomas (NHL) are grouped as nodal and extranodal lymphoma according to their localization. Extranodal localization exists in 25%-40% of all NHL cases and can occur in any organ including gastrointestinal tract, head-neck, orbita, central nervous system, lungs, bones, breast, testis, thyroid and genitourinary system (3). It may be multifocal involving two or more organs (4).

The incidence of secondary renal involvement is about 3% (3). Primary renal lymphoma (PRL) is even more uncommon, accounting for less than 1% of all lymphomas. PRL can occur as a solitary renal nodule or as an infiltrative renal disease. The most common presentation of PRL is acute renal failure, proteinuria, microscopic hematuria and renal enlargement. The prognosis of PRL is usually poor (5). Early diagnosis is important for preserving renal function.

Primary lymphoma of bone is also a rare disease that represents less than 1% of all lymphomas, 5% of extranodal NHL and 3%-5% of all primary bone tumours (6). NHL usually shows solitary bone involvement and mostly involves diametaphysis of long bones (7). The most common manifestation of primary NHL of bone is frequent pathological fracture (8). There are multiple imaging features of primary bone NHL, including a near-normal bone appearance, focal lytic lesion with geographic margins or a diffusely permeative lesion with bone destruction and soft-tissue involvement (9). Although CT scan can discriminate trabecular destruction, periosteal reaction, sequestration and extraosseos lesions in patients with bone involvement, it may not differentiate malign and benign bone tumours. In such cases, more advanced imaging techniques such as magnetic resonance imaging (MRI) and FDG PET/CT imaging are required for diagnosis (10). On the basis of follow-up of patients, the differential diagnosis of persistent lymphoma from healing bone may not be reliably done by MRI but PET/CT imaging has superiority in assessing remission status (11). Shin DS et al. reported that there was low FDG uptake within cortical bone or adjacent soft tissue around the fracture in benign fracture, rarely in the marrow. The patternof intramedullary FDG uptake between malignant and benign fractures were significantly different. The sensitivity, specificity and diagnostic accuracy of F-18 FDG PET/CT were 89.5%, 86.7% and 88.2%, respectively, with a cut-off SUVmax set at 4.7. The time interval between fracture and PET/CT did not significantly influence FDG uptake at the fracture site (12). It has been reported that FDG PET/CT is a superior imaging modality to demonstrate early lymphoma involvement of bones (10). However, FDG PET/CT has some limitations especially in evaluation of minimally residual disease and therapy response in patients with diffuse bone marrow uptake because of growth stimulating factor (GSF) usage (13).

We report an unusual case of extranodal lymphoma of bone and kidneys shown by FDG PET/CT scan. The simultaneous tibia and bilateral renal involvement is rarely seen in lymphoma. This case illustrates the imaging findings of the extranodal DLBCL of kidney and bones and benefits of FDG PET/CT imaging in evaluating the extent of disease and assessing the treatment response in lymphoma.

**Conflicts of Interest**

There are no conflicts of interest.
